# Supplementary material for: Capturing nascent extracellular vesicles by metabolic glycan labeling-assisted microfluidics
Source: Nat Commun. 2023 Oct 17;14:6541. doi: 10.1038/s41467-023-42248-9 (PMC10582105; doi:10.1038/s41467-023-42248-9)
Supplement: Supplementary file 1 — Supplementary Information [file 41467_2023_42248_MOESM1_ESM.pdf]

Supplementary Information

Capturing Nascent Extracellular Vesicles by Metabolic Glycan Labeling-Assisted Microfluidics

Wu *et al.*

Capturing Nascent Extracellular Vesicles by Metabolic Glycan Labeling-Assisted Microfluidics

Qiuyue Wu[1], Wencheng Wang[1], Chi Zhang[1], Zhenlong You[1], Yinyan Zeng[1], Yinzhu Lu[1], Suhui Zhang[1], Xingrui Li[1], Chaoyong Yang[1,2], and Yanling Song[1]*

[1]State Key Laboratory of Physical Chemistry of Solid Surfaces, Key Laboratory for Chemical Biology of Fujian Province, The MOE Key Laboratory of Spectrochemical Analysis & Instrumentation, Department of Chemical Biology, College of Chemistry and Chemical Engineering, Xiamen University, Xiamen 361005, P. R. China.

[2] Institute of Molecular Medicine, Renji Hospital, Shanghai Jiao Tong University School of Medicine, Shanghai, 200127, China
*e-mail: ylsong@xmu.edu.cn

Supplementary Fig. 1 Fluorescence intensity of the MGL A375 EVs treated with DBCO-Cy5.

Supplementary Fig. 2 The intensity ratio of the MGL EVs treated with $Ac_4ManNAz$ versus untreated at different flow rates in Melac-Chip.

Supplementary Fig. 3 Fluorescence intensity of blank sample (without the addition of EVs), MGL EVs (in orange) and non-MGL EVs (in grey) captured by Melac-chip.

Supplementary Fig. 4 Verification of PD-L1 expression on 4T1 cells.

Supplementary Fig. 5 4T1-bearing mouse model with PD-L1 immunotherapy treatment.

Supplementary Fig. 6 Schematics of the detection of (a) nascent $CD63^+$ EVs, (b) nascent PD-$L1^+$ EVs, and (c) total PD-$L1^+$ $CD63^+$ EVs derived from 4T1 cells, as well as the calibration curves.

Supplementary Fig. 7 Schematic of the detection of PD-L1 positive MGL-EVs, as well as the detected intensity of MGL-EVs with anti-PD-L1 blockade (in blue) and without blockade (in grey).

Supplementary Fig. 8 Analyzing of nascent and pre-existing PD-$L1^+$ $CD63^+$ EVs in response to PD-L1 immunotherapy.

Supplementary Fig. 9 B16F10-bearing mouse model with PD-L1 immunotherapy treatment.

Supplementary Fig. 10 Schematics of the detection of (a) nascent PD-$L1^+$ EVs and (b) total PD-$L1^+$ $CD63^+$ EVs derived from B16F10 cells, as well as the calibration curves.

Supplementary Fig. 11 EV analysis for B16F10-bearing mouse model with immunotherapy.

Supplementary Fig. 12 FACS gating strategies.

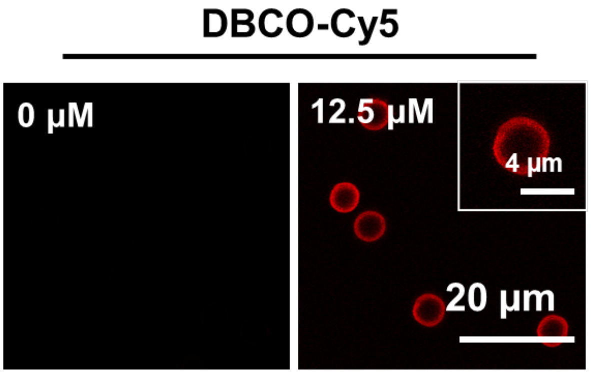

Supplementary Fig. 1 Fluorescence intensity of the MGL A375 EVs treated with DBCO-Cy5. After treating A375 cells with 50 μM $Ac_4ManNAz$ for 60 h, the collected MGL EVs were loaded on latex beads, and then reacted with 12.5 μM DBCO-Cy5 (PBS as the control) for 1 h prior to characterization. MGL refers to metabolic glycan labelling.

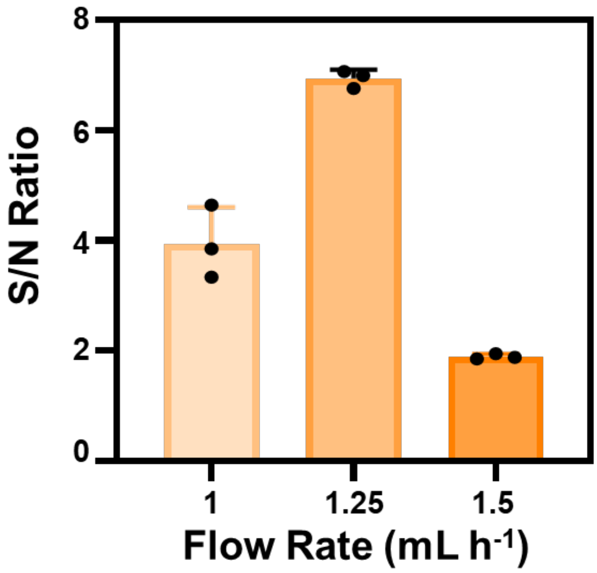

Supplementary Fig. 2 The intensity ratio of the MGL EVs treated with $Ac_4ManNAz$ versus untreated at different flow rates in Melac-Chip. n = 3 biologically independent experiments. Data shown as mean ± SD.

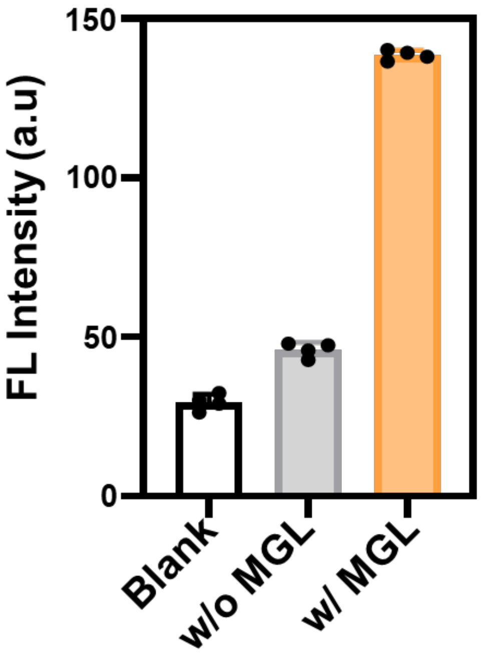

Supplementary Fig. 3 Fluorescence intensity of blank sample (without the addition of EVs), non-MGL EVs (in grey), and MGL EVs (in orange) captured by Melac-Chip. Their fluorescence intensities were 30 (a. u.), 46 (a. u.) and 139 (a. u.), respectively. Therefore, the fluorescence background of non-tagged cell produced EVs is mostly caused by the inherent background of the detection system (such as the autofluorescence of FDG or the chip, tiny non-specific adsorption of antibodies), rather than non-MGL EVs. n = 4 biologically independent experiments. Data shown as mean ± SD.

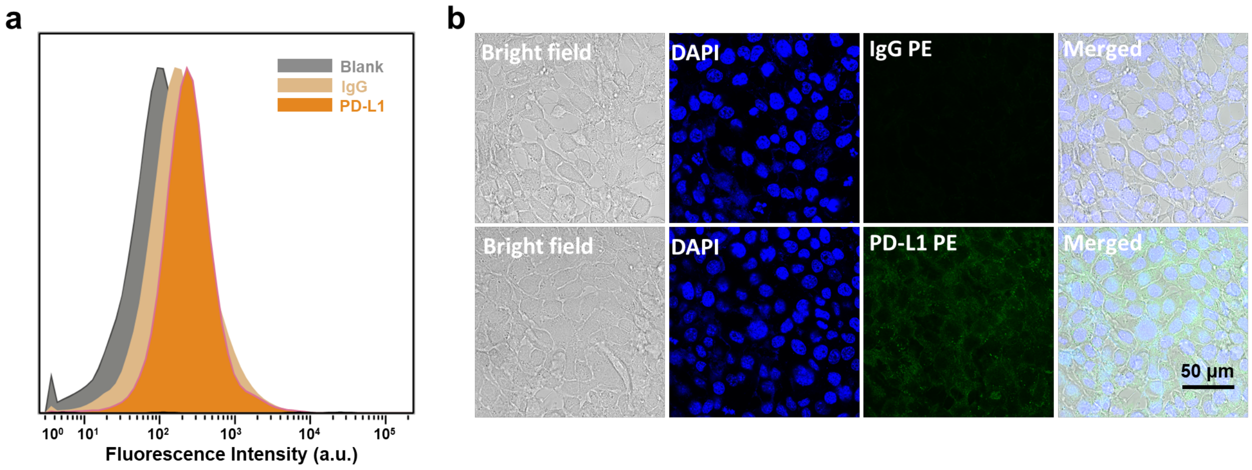

Supplementary Fig. 4 Verification of PD-L1 expression on 4T1 cells. a, Flow cytometry of 4T1 cells by PD-L1 antibody staining. **b,** Confocal laser scanning images of PD-L1 expression on 4T1 cells.

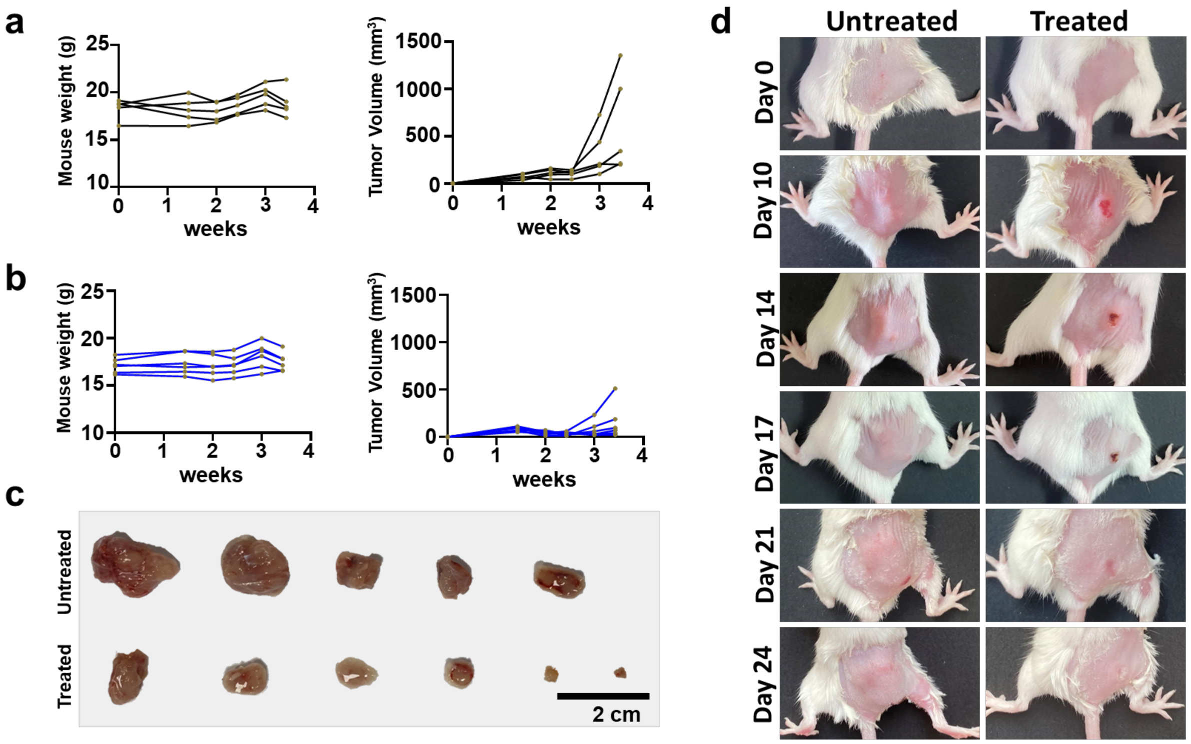

Supplementary Fig. 5 4T1-bearing mouse model with PD-L1 immunotherapy treatment. a-b, The body weight and tumor volume of individual mouse in the (**a**, n = 5 biologically independent experiments) untreated group and (**b**, n = 6 biologically independent experiments) anti-PD-L1 antibody treated group. **c,** The images of tumor tissue from the untreated and treated groups. **d,** Representative images showing the growth of 4T1 tumors in mice with/without PD-L1 antibody immunotherapy.

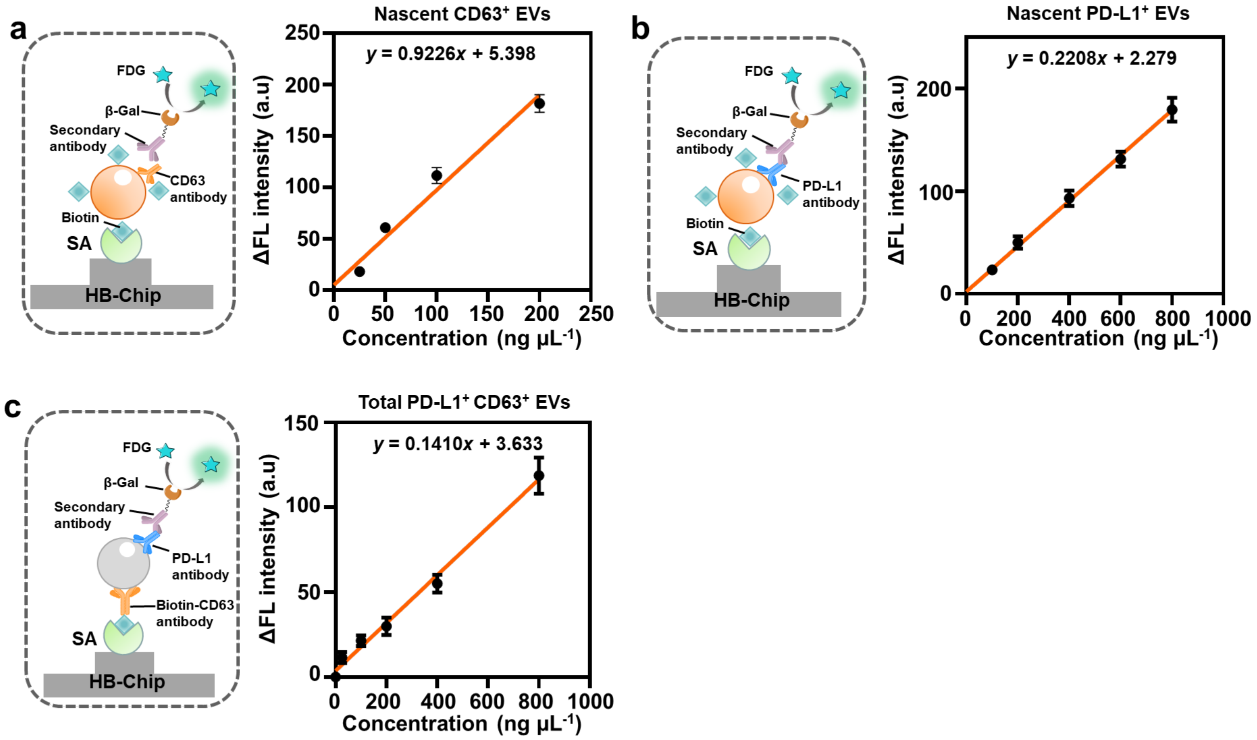

Supplementary Fig. 6 Schematics of the detection of (a) nascent CD63$^+$ EVs, (b) nascent PD-L1$^+$ EVs, and (c) total PD-L1$^+$ CD63$^+$ EVs derived from 4T1 cells, as well as the calibration curves. n =5 biologically independent experiments. Data shown as mean ± SD.

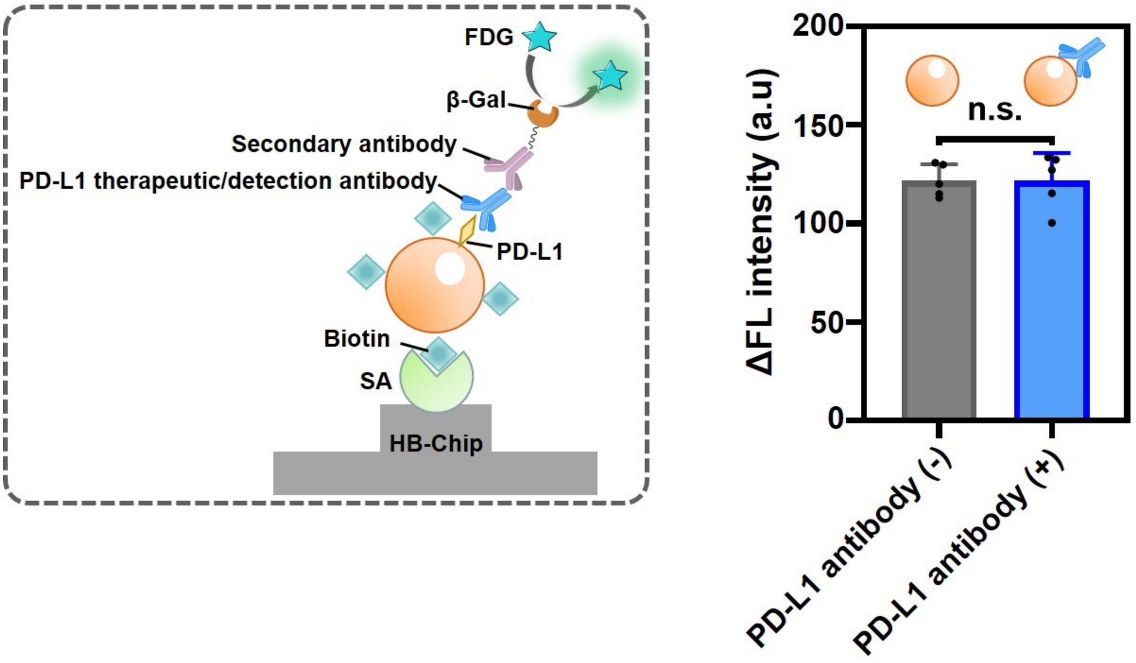

Supplementary Fig. 7 Schematic of the detection of PD-L1 positive MGL-EVs, as well as the detected intensity of MGL-EVs with anti-PD-L1 blockade (in blue) and without blockade (in grey). Because both therapeutic and detectable anti-PD-L1 isotypes were rat IgG, which can be identified by secondary antibody (β-galactosidase-coupled rabbit anti-rat IgG H and L). $\Delta FL = FL - FL_0$, where FL_0 and FL are the fluorescence intensity detected by Melac-Chip before and after the addition of EVs. n = 5 biologically independent experiments. Data shown as mean ± SD.

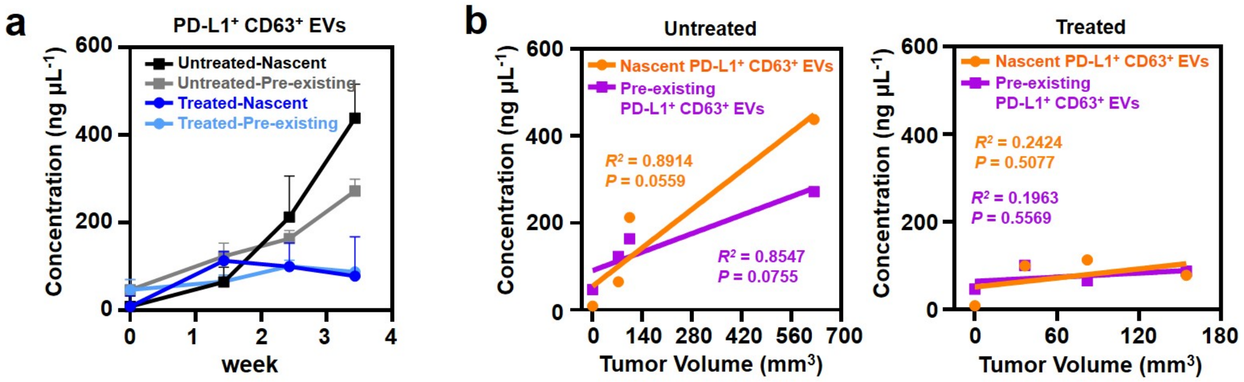

Supplementary Fig. 8 Analyzing nascent and pre-existing PD-L1$^+$ CD63$^+$ EVs in response to PD-L1 immunotherapy. a, The detected concentrations of nascent PD-L1$^+$ CD63$^+$ EVs and pre-existing PD-L1$^+$ CD63$^+$ EVs at different time points. **b,** Pearson correlation of the nascent PD-L1$^+$ CD63$^+$ EVs (orange) and pre-existing PD-L1$^+$ CD63$^+$ EVs (purple) to the tumor volume in 4T1-bearing mice with /without anti-PD-L1 treatment. n = 5 for the untreatment group, n = 6 for the anti-PD-L1 treatment group.

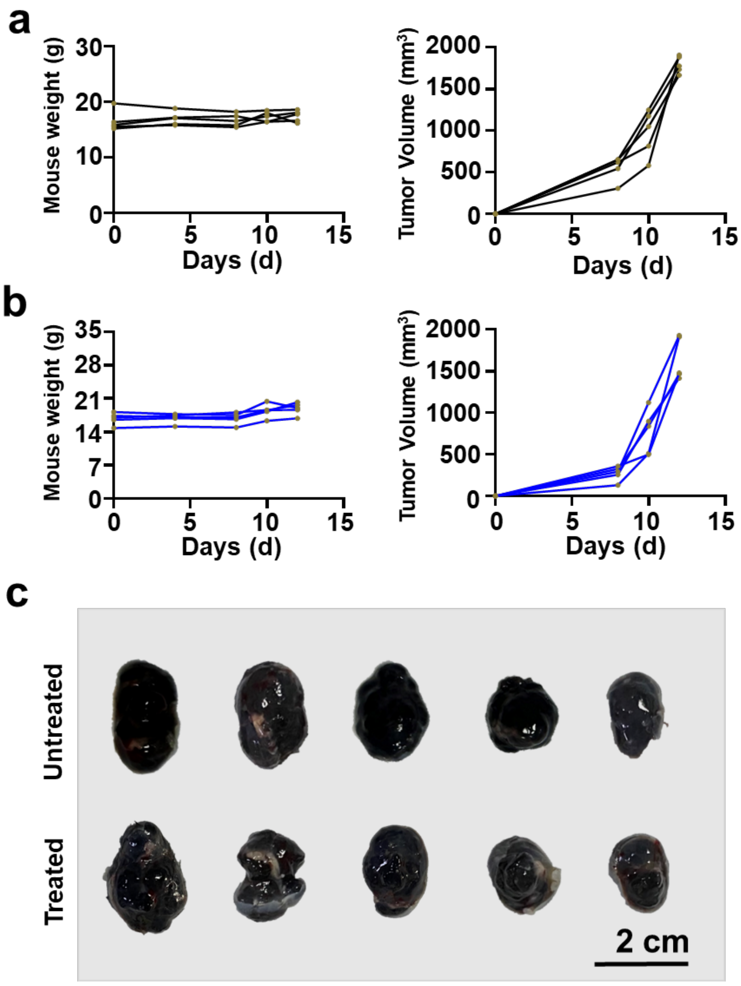

Supplementary Fig. 9 B16F10-bearing mouse model with PD-L1 immunotherapy treatment. a-b, The body weight and tumor volume of individual mouse in the (**a**, n = 5) untreated group, and (**b**, n = 5) anti-PD-L1 antibody treated group. **c,** The images of tumor tissue from the untreated and treated groups.

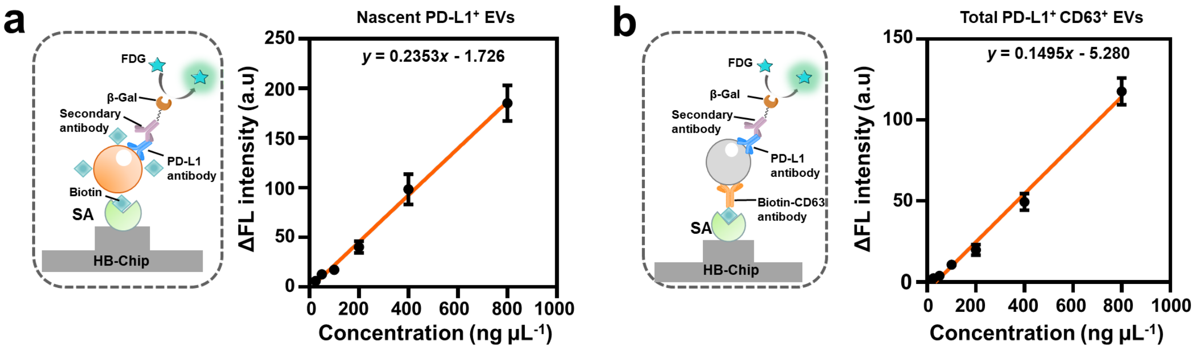

Supplementary Fig. 10 Schematics of the detection of (a) nascent PD-L1^{+} EVs and (b) total PD-L1^{+} CD63^{+} EVs derived from B16F10 cells, as well as the calibration curves. n =5 biologically independent experiments. Data shown as mean ± SD.

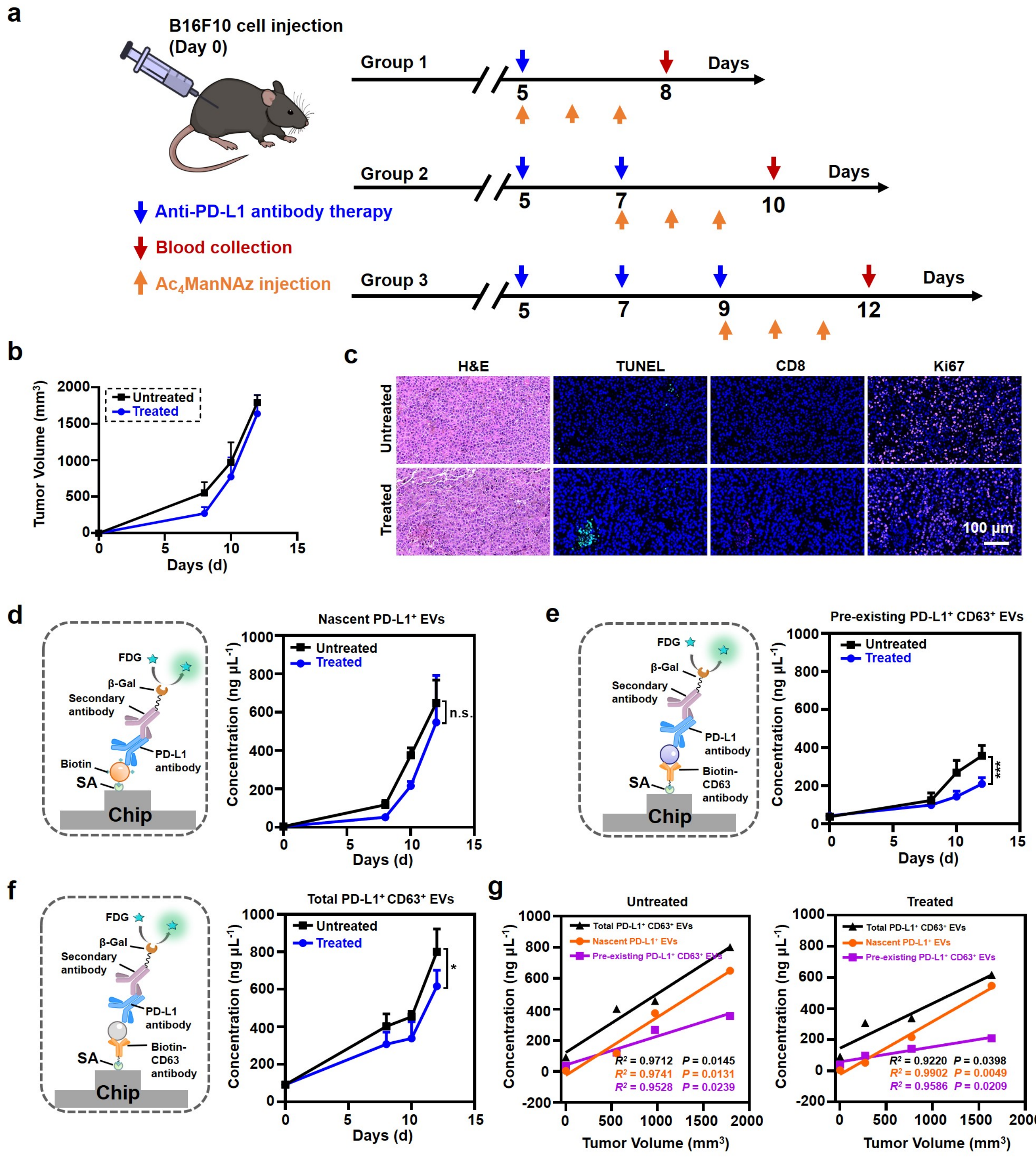

Supplementary Fig. 11 EV analysis for B16F10-bearing mouse model with immunotherapy. a, Schematic of the tumor implantation, PD-L1 immunotherapy, metabolic glycan labelling and sample collection in a B16F10-bearing mouse model. **b,** Tumor growth curves of B16F10-bearing mice with/without PD-L1 antibody treatment. **c,** Representative images of tumor tissue by HE and immunofluorescent staining. HE staining for general histology, TUNEL assay to identify and quantify apoptotic cells, $CD8^+$ T cells to measure tumor infiltration, and Ki-67 protein for tumor cell

proliferation. **d-f,** Schematic of the detection of nascent PD-L1$^+$ EVs (d), pre-existing PD-L1$^+$ CD63$^+$ EVs (e), and total PD-L1$^+$ CD63$^+$ EVs (f) as well as the detected concentrations at different time points. *$P < 0.05$, ***$P < 0.001$, and n.s. indicates non-significant ($P > 0.05$). **g,** Pearson correlation of the nascent PD-L1$^+$ EVs (orange), pre-existing PD-L1$^+$ CD63$^+$ EVs (purple) and total PD-L1$^+$ CD63$^+$ EVs (black) to the tumor volume in B16F10-bearing mice with/without anti-PD-L1 treatment. n = 5 biologically independent experiments. Data shown as mean ± SD.

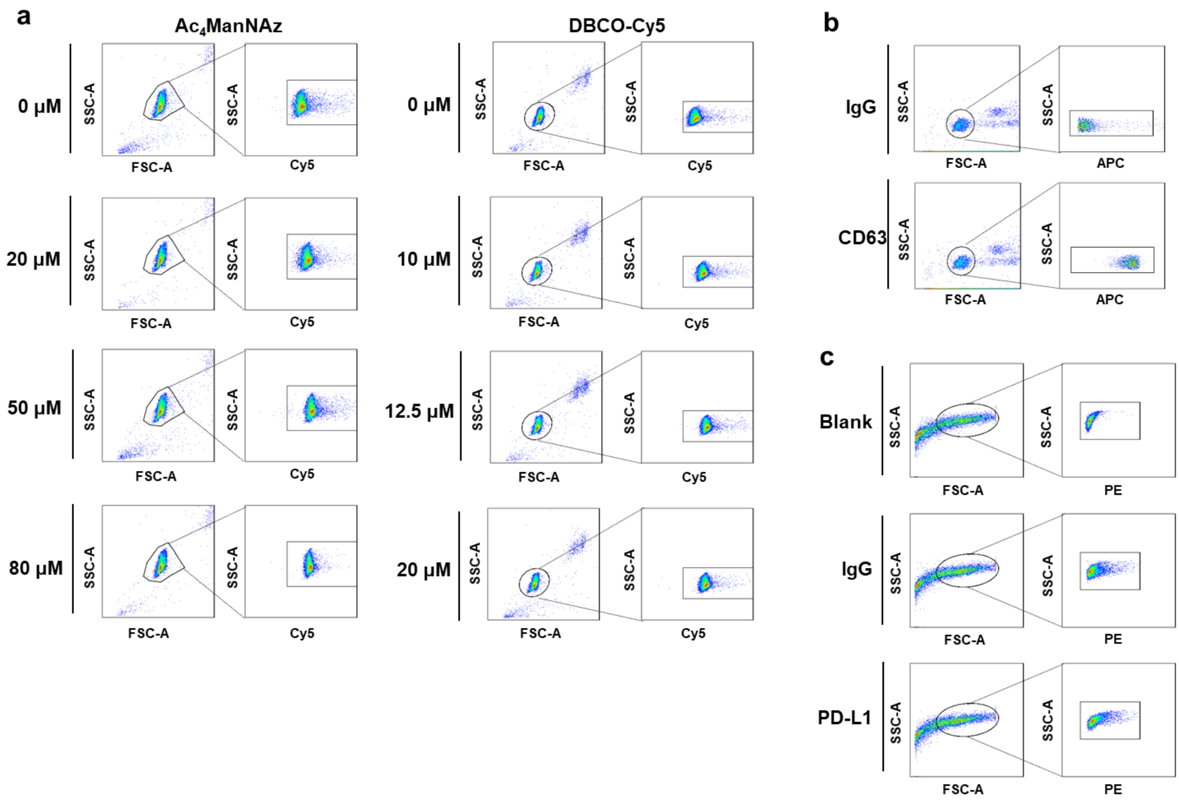

Supplementary Fig. 12 FACS gating strategies. a-b, FACS gating strategies for EVs loaded latex beads, related to Fig. 2b (a) and Fig. 2f (b). **c,** FACS gating strategies for 4T1 cells, related to Supplementary Fig. 5a.
