# Supplementary material for: Capturing nascent extracellular vesicles by metabolic glycan labeling-assisted microfluidics
Source: Nat Commun. 2023 Oct 17;14:6541. doi: 10.1038/s41467-023-42248-9 (PMC10582105; doi:10.1038/s41467-023-42248-9)
Supplement: Supplementary file 2 — Reporting Summary [file 41467_2023_42248_MOESM2_ESM.pdf]

nature portfolio

Corresponding author(s): Yanling Song

Last updated by author(s): Sep 25, 2023

Reporting Summary

Nature Portfolio wishes to improve the reproducibility of the work that we publish. This form provides structure for consistency and transparency in reporting. For further information on Nature Portfolio policies, see our Editorial Policies and the Editorial Policy Checklist.

Statistics

For all statistical analyses, confirm that the following items are present in the figure legend, table legend, main text, or Methods section.

n/a	Confirmed	
☐	☒	The exact sample size (*n*) for each experimental group/condition, given as a discrete number and unit of measurement
☐	☒	A statement on whether measurements were taken from distinct samples or whether the same sample was measured repeatedly
☐	☒	The statistical test(s) used AND whether they are one- or two-sided<br>*Only common tests should be described solely by name; describe more complex techniques in the Methods section.*
☐	☒	A description of all covariates tested
☐	☒	A description of any assumptions or corrections, such as tests of normality and adjustment for multiple comparisons
☐	☒	A full description of the statistical parameters including central tendency (e.g. means) or other basic estimates (e.g. regression coefficient) AND variation (e.g. standard deviation) or associated estimates of uncertainty (e.g. confidence intervals)
☐	☒	For null hypothesis testing, the test statistic (e.g. *F*, *t*, *r*) with confidence intervals, effect sizes, degrees of freedom and *P* value noted<br>*Give P values as exact values whenever suitable.*
☒	☐	For Bayesian analysis, information on the choice of priors and Markov chain Monte Carlo settings
☐	☒	For hierarchical and complex designs, identification of the appropriate level for tests and full reporting of outcomes
☐	☒	Estimates of effect sizes (e.g. Cohen's *d*, Pearson's *r*), indicating how they were calculated

Our web collection on statistics for biologists contains articles on many of the points above.

Software and code

Policy information about availability of computer code

Data collection	Thermo Xclibur 2.2 SP1.48; NIS-Elements Basic Research; BD FACSuite
Data analysis	Statistical analysis was analyzed by GraphPad Prism 8.3 and Microsoft Excel. LC-MS/MS analysis was performed on an Orbitrap Fusion™ Lumos™ Tribrid mass spectrometer with an EASY-IC ion source (Thermo Scientific Inc, US) and the raw data was analyzed by Proteome Discoverer 2.2 software (Thermo Scientific Inc, US) against uniprot database, and gene ontology analysis was analyzed by the Database for Annotation, Visualization and Integrated Discovery (DAVID) (https://david.ncifcrf.gov/home.jsp). Confocal microscopy imaging of CD63 antibody satined beads and DiI-stained MGL EVs captured by the chip was performed on a TCS SP8-STED 3X microscope (Leica), and raw data was analyzed by Leica Application Suite 4.0. Fluorescence microscopic imaging of on-chip fluorescence enzyme immunoassay was performed on NIS-Elements Basic Research Software, and raw data was analyzed by Image J (version 1.52a). The images of hematoxylin-eosin staining were analyzed by Motic DSAssistant Lite 1.0. The images of immunofluorescence staining were analyzed by Case Viewer2.4. The fluorescence intensities on beads were measured by flow cytometry (BD FACSVerse™) and analyzed by BD FACSuite Flow Cytometry software.

For manuscripts utilizing custom algorithms or software that are central to the research but not yet described in published literature, software must be made available to editors and reviewers. We strongly encourage code deposition in a community repository (e.g. GitHub). See the Nature Portfolio guidelines for submitting code & software for further information.

Data

Policy information about availability of data

All manuscripts must include a data availability statement. This statement should provide the following information, where applicable:

- Accession codes, unique identifiers, or web links for publicly available datasets
- A description of any restrictions on data availability
- For clinical datasets or third party data, please ensure that the statement adheres to our policy

The data supporting the findings of this study are available within the paper and its Supplementary Information files. Should any raw data files be needed in another format they are available from the corresponding author upon reasonable request. Source data are provided with this paper.

Research involving human participants, their data, or biological material

Policy information about studies with human participants or human data. See also policy information about sex, gender (identity/presentation), and sexual orientation and race, ethnicity and racism.

Reporting on sex and gender	N/A
Reporting on race, ethnicity, or other socially relevant groupings	N/A
Population characteristics	N/A
Recruitment	N/A
Ethics oversight	N/A

Note that full information on the approval of the study protocol must also be provided in the manuscript.

Field-specific reporting

Please select the one below that is the best fit for your research. If you are not sure, read the appropriate sections before making your selection.

☒ Life sciences ☐ Behavioural & social sciences ☐ Ecological, evolutionary & environmental sciences

For a reference copy of the document with all sections, see nature.com/documents/nr-reporting-summary-flat.pdf

Life sciences study design

All studies must disclose on these points even when the disclosure is negative.

Sample size	Sample sizes for in vivo experiments were used on the basis of ensuring results obtained were of a representable quantity. A population range of 3-6 mice per group was used to ensure statistical power. For in vitro studies, sample size of at least 3 was used in each experiment. Sample size and number of independent experiments are stated in the figure legends.
Data exclusions	No data were excluded.
Replication	All tests were repeated at least three times with consistent results.
Randomization	Animals were randomly allocated to different groups before treatment. Cells and other samples were also randomly allocated into different groups before treatment.
Blinding	All data are unbiasedly collected and analysed. The investigatiors were not blinded to allocation during experiments and outcome assessment.

Reporting for specific materials, systems and methods

We require information from authors about some types of materials, experimental systems and methods used in many studies. Here, indicate whether each material, system or method listed is relevant to your study. If you are not sure if a list item applies to your research, read the appropriate section before selecting a response.

Materials & experimental systems

n/a	Involved in the study	
☐	☒	Antibodies
☐	☒	Eukaryotic cell lines
☒	☐	Palaeontology and archaeology
☐	☒	Animals and other organisms
☒	☐	Clinical data
☒	☐	Dual use research of concern
☒	☐	Plants

Methods

n/a	Involved in the study	
☒	☐	ChIP-seq
☐	☒	Flow cytometry
☒	☐	MRI-based neuroimaging

Antibodies

Antibodies used

Anti-human CD63 APC antibody (Thermo Fisher Scientific Inc, Cat#A15712, 10 µL/sample)
IgG-APC (Santa Cruz, Texas, USA, Cat#sc-516612,10 µL/sample).
Anti-mouse CD63 antibody (R&D systems, USA, Cat#MAB5417, 20 µg/mL)
Biotinylated anti-mouse CD63 antibody (Bioss, Beijing, China, Cat#bs-23032R-Bio, 20 µg/mL)
Mouse anti-Human CD63 (BD Pharmingen, USA, Cat#556019, 20 µg/mL)
Anti-PD-L1 antibody (Novus Biologicals, USA, Cat#NBP1-43262, 20 µg/mL)
Goat anti-mouse IgG H&L beta-galactosidase (Abcam, USA, Cat#ab136775, 120 µg/mL)
Rabbit Anti-Rat IgG H&L beta-galactosidase) (Abcam, USA, Cat#ab136716, 120 µg/mL)
Anti-mouse PD-L1 immunotherapeutic antibody (BioXCell, USA, Cat#BE0101, 12.5 mg/kg)

Validation

All antibodies used in our research were commercially available and validated. Any additional information regarding the validation performed by the manufacturer can be retrieved from their website.
Anti-human CD63 APC antibody (Cat#A15712): https://www.thermofisher.cn/cn/zh/antibody/product/CD63-Antibody-Monoclonal/A15712
IgG-APC (Cat#sc-516612): https://www.scbt.com/p/normal-mouse-igg-apc?requestFrom=search
Anti-mouse CD63 antibody (Cat#MAB5417): https://www.rndsystems.com/cn/products/mouse-cd63-antibody-446703_mab5417
Biotinylated anti-mouse CD63 antibody (Cat#bs-23032R-Bio): http://www.biosschina.com/#/productDetail?goods_id=97758
Mouse anti-Human CD63 (Cat#556019): https://www.bdbiosciences.com/en-us/products/reagents/flow-cytometry-reagents/research-reagents/single-color-antibodies-ruo/purified-mouse-anti-human-cd63.556019
Anti-PD-L1 antibody (Cat#NBP1-43262): https://www.novusbio.com/products/pd-l1-antibody-mih5_nbp1-43262
Goat anti-mouse IgG H&L (beta-galactosidase) (Cat#ab136775): https://www.abcam.cn/products/secondary-antibodies/goat-mouse-igg-hl-beta-galactosidase-ab136775.html
Rabbit Anti-Rat IgG H&L (beta-galactosidase) (Cat#ab136716): https://www.abcam.cn/products/secondary-antibodies/rabbit-rat-igg-hl-beta-galactosidase-ab136716.html
Anti-mouse PD-L1 antibody (Cat#BE0101): https://bioxcell.com/invivomab-anti-mouse-pd-l1-b7-h1-be0101

Eukaryotic cell lines

Policy information about cell lines and Sex and Gender in Research

Cell line source(s)

Human melanoma A375 (#CRL-1619) cells were purchased from American Type Culture Collection (ATCC). Murine breast cancer 4T1 cell lines (#CL-0007) and B16F10 mouse melanoma cells (#CL-0319) were purchased from Procell Life Science & Technology Co.,Ltd. (Wuhan, China).

Authentication

All cell lines have been authenticated using short tandem repeat (STR) profiling.

Mycoplasma contamination

All cell lines were tested negative for mycoplasma contamination.

Commonly misidentified lines
(See ICLAC register)

No misidentified cell lines were used in this study.

Animals and other research organisms

Policy information about studies involving animals; ARRIVE guidelines recommended for reporting animal research, and Sex and Gender in Research

Laboratory animals

Female BALB/c strains and female C57BL/6J strains of mice at 6-8 weeks of age were purchased from Xiamen University Laboratory Animal Center. All mice were housed in Animal Care Center of Xiamen University (at 20–24 °C, relative humidity of 40–60%, a 12 h light/dark cycle).

Wild animals

No wild animal was used in this study.

Reporting on sex

Female animals were used in this study.

Field-collected samples	No field collected samples were involved in this study.
Ethics oversight	All animal studies were conducted in accordance with the National Institute Guide for the Care and Use of Laboratory Animals. The experimental protocols (XMULAC20220298) were approved by the Institutional Animal Care and Use Committee (IACUC) of Xiamen University.

Note that full information on the approval of the study protocol must also be provided in the manuscript.

Flow Cytometry

Plots

Confirm that:

- [x] The axis labels state the marker and fluorochrome used (e.g. CD4-FITC).
- [x] The axis scales are clearly visible. Include numbers along axes only for bottom left plot of group (a 'group' is an analysis of identical markers).
- [x] All plots are contour plots with outliers or pseudocolor plots.
- [x] A numerical value for number of cells or percentage (with statistics) is provided.

Methodology

Sample preparation	General procedure of flow cytometry analysis of EVs:<br>Ten μg EVs were mixed with 4 μL aldehyde/sulfate latex beads for 15 min adsorption at room temperature. The EV-bead complexes were then blocked by 100 μL PBS with 1 M glycine and 20% BSA for 30 min. After washing twice by PBS with 0.5% BSA, the beads were pelleted by centrifugation (3,968 g, 5 min) (Eppendorf, Centrifuge 5424R), and resuspended in 40 μL PBS with 0.5% BSA. Four μL of these EV-modified beads were incubated with anti-CD63 antibody (IgG as a control) for 1 hour. After washing twice by PBS buffer with 0.5% BSA, the fluorescence intensities were measured by flow cytometry (BD FACSVerse™). The acquired data of flow cytometry were analyzed by BD FACSuite Flow Cytometry software.
Instrument	BD FACSVerse™
Software	BD FACSuite Flow Cytometry Software
Cell population abundance	The target populations (P1) were isolated by gating on fluorescence intensity. E.g. ~70% of control sample were at P1, while ~75% of CD63-APC treated sample.
Gating strategy	Target populations were distinguished from whole scatter via FSC-A × SSC-A gating. Gating on FSC-A/SSC-A was used to eliminate bead aggregation and ensure the collection of only single type.

- [x] Tick this box to confirm that a figure exemplifying the gating strategy is provided in the Supplementary Information.
